# Schnitt-Naht-Zeiten bei der endoskopischen Ohrchirurgie

**DOI:** 10.1007/s00106-021-01066-5

**Published:** 2021-06-14

**Authors:** S. Preyer

**Affiliations:** Klinik für Hals-Nasen-Ohrenheilkunde, Kopf- und Halschirurgie und plastische Gesichtschirurgie, ViDia-Kliniken Karlsruhe, Steinhäuserstraße 18, 76133 Karlsruhe, Deutschland

**Keywords:** Videoassistierte Chirurgie, Minimal-invasive Operationsverfahren, Operationsdauer, Otitis media, Mittelohr, Video-assisted surgery, Minimally invasive surgical procedures, Operative time, Otitis media, Middle ear

## Abstract

**Hintergrund:**

Endoskopische Ohrchirurgie („endoscopic ear surgery“, EES) wird international immer häufiger anstelle der mikroskopischen Ohrchirurgie („microscopic ear surgery“, MES) eingesetzt, hat sich in Deutschland aber als Routineverfahren noch nicht etabliert.

**Fragestellung:**

Untersucht wurden die Schnitt-Naht-Zeiten bei der EES und die Praktikabilität der Methode im deutschen Klinik-Setting.

**Material und Methoden:**

In einer retrospektiven Studie wurden 60 konsekutive mikroskopisch operierte Patienten (MES) von 2015 mit 60 konsekutiven endoskopisch operierten Patienten aus dem Jahr 2018 verglichen. Verglichen wurden Hörergebnisse, Trommelfellbefund und Schnitt-Naht-Zeiten nach 3 Wochen.

**Ergebnisse:**

Bei endoskopisch geführten Ohroperationen war der Zugang meistens transmeatal und musste seltener als bei mikroskopisch durchgeführten Operationen die Gehörgangsvorderwand zurückgeschliffen werden. Die Operationszeiten unterschieden sich in den 2 Gruppen statistisch nicht signifikant. Ein Trommelfellverschluss gelang bei 57/60 Patienten in der mikroskopischen Gruppe und bei 59/60 in der endoskopischen Gruppe. Eine Hörverbesserung wurde in beiden Gruppen erreicht. Die Hörverbesserung war in den 2 Gruppen statistisch signifikant nicht unterschiedlich.

**Schlussfolgerungen:**

Endoskopische Ohrchirurgie ist eine zeitökonomische und minimal-invasive Methode und stellt bei vergleichbaren Ergebnissen eine praktikable Alternative zur mikroskopischen Ohrchirurgie dar.

## Abstract (en)

**Background:**

Endoscopic ear surgery (EES) is increasingly used internationally instead of microscopic ear surgery (MES), but has not yet become established as a routine procedure everywhere in Germany.

**Objective:**

The incision–suture time and practicability of EES in the setting of a German clinic were investigated.

**Materials and methods:**

In a retrospective study, 60 consecutive MES patients from 2015 were compared with 60 consecutive EES patients from 2018. Hearing results, tympanic membrane findings after 3 weeks, and incision–suture times were compared.

**Results:**

In EES, access was mostly transmeatal and bone of the anterior auditory canal wall had to be removed less often than in MES. There was no statistically significant difference in surgery times between the two groups. Perforation closure was achieved in 57/60 and 59/60 ears in the MES and EES groups, respectively. Hearing was improved in both groups. There was no statistical difference in hearing improvement between the two groups.

**Conclusion:**

EES is a time efficient and minimally invasive method which represents a viable alternative to MES with comparable results.

Die endoskopische Ohrchirurgie („endoscopic ear surgery“, EES) stellt eine Weiterentwicklung und Verfeinerung, sowie Ergänzung der mikroskopischen Mittelohrchirurgie („microscopic ear surgery“, MES) dar [2, 5, 6, 10, 11]. Die Visualisierung der feinen Mittelohrstrukturen erfolgt über ein starres Endoskop anstelle des Operationsmikroskops. Der Operateur verfolgt die Operation auf einem Monitor, auf den das Endoskopbild über eine Kamera (vorzugsweise HD oder 4k) projiziert wird. Der Monitor wird normalerweise in Bezug auf die Position des Operateurs auf der gegenüberliegenden Seite des Patientenkopfs platziert [9]. Bei normaler Gehörgangsweite erfolgt die Operation transmeatal ohne äußerlichen Schnitt. Das Endoskop wird im äußeren Gehörgang platziert und die Instrumente über den intakten äußeren Gehörgang nach Bildung eines verkürzten tympanomeatalen Lappens in das Mittelohr eingeführt. Lediglich bei der Gewinnung von Gewebetransplantaten kann ein Schnitt, z. B. an der Tragus-Innenseite, für Knorpel-Perichondrium, oder retroaurikulär, für die Fasziengewinnung, notwendig werden. Zur Vermeidung eines Schnitts verwenden einige Chirurgen kommerziell erhältliche Biodesign-Transplantate [3].

In Deutschland hat sich die endoskopische Ohrchirurgie noch nicht als Routinemethode durchgesetzt. Ziel der retrospektiven Studie war eine Abschätzung der Machbarkeit und Praktikabilität der endoskopischen Ohroperationstechnik in der Mittelohrchirurgie im Vergleich zum ausschließlich mikroskopischen Vorgehen im Umfeld einer deutschen Hals-Nasen-Ohren-Klinik mit hohen Hygienestandards, starkem Kostendruck und knappen Zeitressourcen.

## Methodik

Eine Fallserie von 60 konsekutiven EES-Eingriffen zwischen 6/2017 und 4/2018 wurde mit einem MES-Patientenkollektiv, bestehend aus 60 konsekutiven Fällen, zwischen 1/2015 und 9/2015 verglichen. Bei den endoskopischen Eingriffen handelte es sich um Typ-2b- oder -3-EES-Eingriffe, klassifiziert nach Cohen et al. [1], d. h., das Endoskop wurde mehr als 50 bzw. 100 % der Operationsdauer zur Visualisierung eingesetzt. EES-Operationen mit Einsatz des Endoskops unter 50 % der Schnitt-Naht-Zeit (Cohen Typ 2a) oder Verwendung des Endoskops ausschließlich zur Inspektion (Cohen Typ 1) wurden nicht in die Studie eingeschlossen [1]. Alle Operationen wurden von der Autorin selbst mit > 30 Jahren Erfahrung in der mikroskopischen Ohrchirurgie und > 30 Jahren Erfahrung in der endoskopischen Nasennebenhöhlenchirurgie durchgeführt. In die Studie wurden keine Stapesplastiken, keine Cholesteatome mit ausgedehntem Mastoidbefall oder Mittelohrtumoren aufgenommen.

Die verwendeten Endoskope hatten eine Länge von 11 cm mit einem Außendurchmesser von 3 mm; eingesetzt wurden 0°-, 30°- und 45°-Winkeloptiken (Fa. Spiggle & Theis Medizintechnik, Overath).

Die Altersverteilung der 120 analysierten Fälle war in beiden Fallserien mit Peaks zwischen dem 10.–20. und 60.–80. Lebensjahr sehr ähnlich. Auch die Geschlechtsverteilung ähnelte sich in den 2 Gruppen mit 25 weiblichen und 35 männlichen Patienten in der EES-Gruppe bzw. 27 weiblichen und 33 männlichen Patienten in der MES-Serie. Die Seitenverteilung war unterschiedlich: Es wurden in der EES-Gruppe 26 rechte und 34 linke Ohren operiert, in der MES-Gruppe waren es 37 rechte und 23 linke Ohren. Die Tab. 1 unterteilt die 2 Patientenkollektive in 4 Untergruppen: Tympanoskopien, sanierende Operationen bei Mittelohradhäsivprozess/kleine Cholesteatome sowie Tympanoplastiken nach Wullstein Typ 1 und 3. [16]. Zur detaillierten Beschreibung der chirurgischen Vorgehensweise wurden die Operationsberichte retrospektiv nach SAMEO-ATO klassifiziert (System zur Einteilung von Mastoidoperationen, SAMEO, und Mittelohroperationen, ATO, Akronym: „stage of operation, approach, mastoidectomy procedure, external auditory canal reconstruction, obliteration of mastoid cavity“; „access, tympanic membrane repair, ossicular chain repair“; Tab. 2; [17]). Hier zeigt sich, dass in der EES-Gruppe 15 Tympanoplastikrevisionen erfolgten, davon 11 geplante (z. B. Second-Look-Eingriffe, S2p), dagegen waren es 22 Revisionen in der MES-Gruppe mit 20 ungeplanten Revisionsoperationen (S2r).

**Table 1:** 

	EES	MES
Tympanoplastik Typ 1	16	23
Tympanoplastik Typ 3	16	18
Tympanoskopie	9	7
Cholesteatom/Adhäsivprozess	19	12

Die 60 Ohroperationen in jeder Patientengruppe setzten sich zusammen aus: Tympanoplastiken Typ 1 oder 3 nach der Wullstein-Klassifikation, Tympanoskopien bei akuter Ertaubung und Operationen bei Cholesteatomen oder Adhäsivprozess

*EES* endoskopische Ohrchirurgie („endoscopic ear surgery“), *MES *mikroskopische Mittelohrchirurgie („microscopic ear surgery“)

**Table 2:** 

SAMEO	EES	MES	ATO	EES	MES
S1	45	37	Ax	53	24
S2p	11	2	A1	0	4
S2r	4	20	A2	7	29
A1	55	0	A3	0	2
A2	0	8	Tx	10	7
A3	3	13	T1	0	0
A4	1	38	T2	46	48
Mx	54	35	T3	4	4
M1a	0	2	On	44	41
M1b	0	0	Ox	0	0
M2a	5	12	Osm	1	1
M2b	1	0	Ost	8	8
M2c	0	0	Osd	2	3
E2	6	10	Ofi	1	1
O	0	0	Oft	4	4
			Ofm	0	1

Die Ohroperationen der 120 Patienten wurden retrospektiv nach der internationalen SAMEO-ATO-Klassifizierung anhand der Operationsberichte klassifiziert

*EES* endoskopische Ohrchirurgie („endoscopic ear surgery“), *MES* mikroskopische Mittelohrchirurgie („microscopic ear surgery“), *SAMEO-ATO* System zur Einteilung von Mastoidoperationen, SAMEO, und Mittelohroperationen, ATO, Akronym: „stage of operation, approach, mastoidectomy procedure, external auditory canal reconstruction, obliteration of mastoid cavity“; „access, tympanic membrane repair, ossicular chain repair“

Der postoperative Trommelfellbefund wurde ohrmikroskopisch 3 Wochen nach der Operation am Tag der Tamponadeentfernung erhoben. Die Nachbetreuung der Patienten nach der Detamponade erfolgte i. d. R. durch die niedergelassenen Hals-Nasen-Ohren-Ärzte der Patienten.

Die präoperativen Hörschwellen wurden an der Klinik der Autorin innerhalb von einer Woche vor der Operation gemessen. Dagegen stammten die meisten postoperativen Hörbefunde aus den Praxen der niedergelassenen Hals-Nasen-Ohren-Ärzte der Patienten. An der Klinik der Autorin wird bei der Detamponade üblicherweise keine Hörprüfung durchgeführt, sondern die Durchführung eines Hörtests 3 Wochen nach der Detamponade bzw. nach Abheilung des Ohrs empfohlen. In der EES-Gruppe wurde bei 6 Patienten kein postoperatives Tonaudiogramm angefertigt; in der MES-Gruppe gab es bei 5 Patienten kein postoperatives Tonaudiogramm, bei einem Patienten ein Tonaudiogramm, bei dem nur die Luftleitung gemessen wurde. Dies war darauf zurückzuführen, dass Patienten entweder nicht mehr zu ihrem Hals-Nasen-Ohren-Arzt zurückkehrten oder die postoperative Messung der Hörschwelle ablehnten.

## Ergebnisse

### Zugang zum Mittelohr

**Figure 1:**
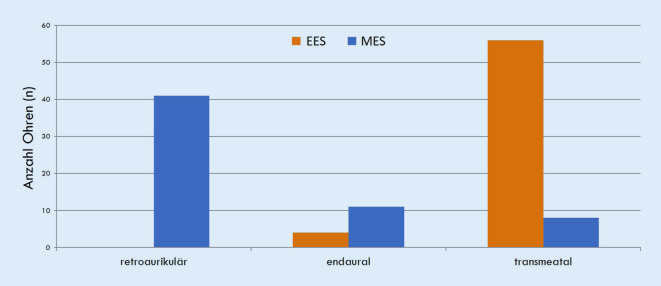

Die Abb. 1 zeigt eindrücklich, dass sich der Zugang zum Mittelohr bei den 2 Techniken stark unterscheidet. Bei der EES war der favorisierte Zugang transmeatal (A1 = 55), während bei der MES eher ein retroaurikulärer Zugang gewählt wurde (A4 = 38; Abb. 1 ). Obwohl der retroaurikuläre Zugang den Einblick in den vorderen tympanomeatalen Winkel im Vergleich zum transmeatalen Zugang oder endauralen Schnitt eher erleichtert, war es bei 14 von 23 Fällen der MES-Tympanoplastiken Typ 1 notwendig, die vordere Gehörgangswand zurückzuschleifen (entspricht 61 %). Dagegen wurde die vordere Gehörgangswand bei 16 EES-Tympanoplastiken Typ 1 nur in 3 Fällen abgetragen (entspricht 18 %), obwohl ein transmeataler Zugang gewählt wurde. Auch bei Betrachtung der Gesamtkollektive mit der ATO-Klassifikation wurde in der EES-Gruppe der Gehörgang in 53/60 Fällen (Ax) (88 %) nicht bearbeitet. Dagegen fand in der MES-Gruppe nur in 24/60 Fällen (40 %) keine Gehörgangserweiterung statt (Ax) und wurde der Gehörgang in 33 Fällen operativ geweitet (A1 und A2). Dies entspricht der Erfahrung, dass mit den modernen Weitwinkeloptiken die Übersicht über das Trommelfell und die Mittelohrstrukturen deutlich besser als mit dem Mikroskop ist. Während bei der MES häufig eine überhängende vordere Gehörgangswand den Blick auf den vorderen tympanomeatalen Winkel optisch verlegt, ist bei der EES eine Knochenabtragung i. d. R. nur erforderlich, wenn vorspringender Knochen mechanisch verhindert, dass die pathologische Veränderung im Mittelohr mit den Instrumenten erreicht wird; die Sicht auf die pathologische Veränderung ist normalerweise kein Problem.

### Schnitt-Naht-Zeiten

**Figure 2:**
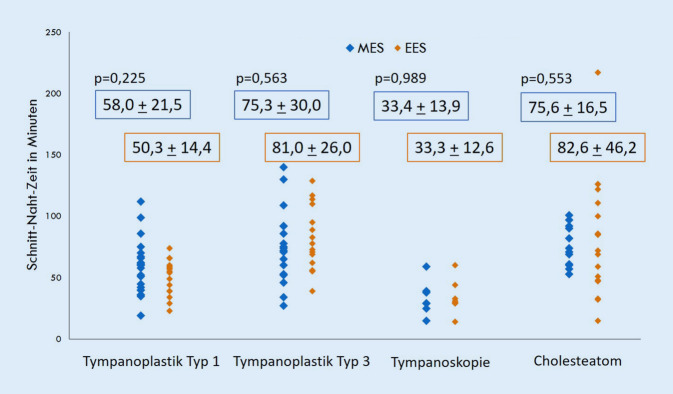

Ein Nachteil der EES ist das einhändige Arbeiten, da die nicht dominante Hand des Operateurs das Endoskop führt. Dies könnte theoretisch zu längeren Operationszeiten führen. Es wurden daher die Schnitt-Naht-Zeiten der 2 Fallserien miteinander verglichen (Abb. 2). Obwohl die Operateurin zum Zeitpunkt der Studie 30 Jahre MES-Erfahrung und nur 3 Jahre EES-Erfahrung hatte, unterschieden sich die Schnitt-Naht-Zeiten bei der Tympanoplastik Typ 1 und 3 sowie bei der Tympanoskopie nicht signifikant voneinander. Lediglich bei der sanierenden Ohrchirurgie, beim Cholesteatom und Adhäsivprozess, zeigt die EES eine sehr breite Streuung und im Mittelwert längere Operationszeiten (82,6 ± 46,2 min), die aber statistisch nicht signifikant (*p* = 0,553) länger als in der MES-Gruppe (75,6 ± 16,5 min) waren.

### Hörergebnisse

Die Knochenleitung prä- und postoperativ blieb in der MES-Gruppe gleich, während in der EES-Gruppe eine diskrete, statistisch signifikante (*p* = 0,008) Verbesserung der Knochenleitungsschwelle von −2 dB HL gemessen wurde (Tab. 3). Die Knochenleitungs-Luftleitungs-Differenz („air-bone gap“, ABG) war in beiden Gruppen postoperativ signifikant geringer als präoperativ. Es gab weder einen statistisch signifikanten Unterschied der Reduktion der ABG (∆ ABG) zwischen den gesamten MES- und EES-Gruppen noch bei Betrachtung der Unterpopulation der Fälle, bei denen eine Gehörknöchelchenkettenrekonstruktion (∆ ABG Typ 3) erfolgte. Bei Berechnung des Hörverlusts nach Röser 1973 [11] prä- und postoperativ ergab sich für beide Gruppen eine mittlere Hörverbesserung von −7 % ohne statistisch signifikanten Unterschied (*p* = 0,981) zwischen den 2 Gruppen (Tab. 3).

**Table 3:** 

	MES prä	t‑Test-*p*	MES post	EES prä	t‑Test-*p*	EES post
KL	20 ± 17,6	0,221	19 ± 18,2	24 ± 24,0	0,008	22 ± 23,9
ABG	17 ± 14,1	0,005	13 ± 12,1	17 ± 19,8	0,026	14 ± 18,7
HV (Rö 73)	38 ± 26,9	0,008	31 ± 28,1	37 ± 29,0	0,003	30 ± 28,9
	MES	t‑Test-*p*	EES			––
∆ ABG prä-post	−5 ± 11,6	0,235	−3 ± 9,0	–	–	–
∆ ABG Typ 3 prä-post	−9 ± 12,6	0,172	−3 ± 9,9	–	–	–
∆ HV (Rö 73) prä-post	−7 ± 17,7	0,981	−7 ± 15,6	–	–	–

Für die Berechnung der statistischen Signifikanz wurde in allen Fällen im doppelseitigen t‑Test ein Signifikanzniveau von *p* = 0,05 angenommen

*EES* endoskopische Ohrchirurgie („endoscopic ear surgery“), *MES *mikroskopische Mittelohrchirurgie („microscopic ear surgery“), *KL* Knochenleitungsschwelle in dB HL, *ABG* „air-bone gap“, Knochenleitungs-Luftleitungs-Differenz in dB HL, *HV* Hörverlust in Prozent nach Röser 1973 [11], berücksichtigt werden die Frequenzen 0,5; 1; 2 und 4 kHz, *Prä* präoperativ, *post* postoperativ, *∆ ABG* und *∆ HV* Unterschied zwischen prä- und postoperativem Befund für Knochenleitungs-Luftleitungs-Differenz und Hörverlust nach Röser 1973 [11], *∆ ABG Typ3* Unterschied der Knochenleitungs-Luftleitungs-Differenz prä- und postoperativ nur für die Untergruppe der Tympanoplastiken Typ 3

### Trommelfellverschluss

Zum Zeitpunkt der Detamponade lag in der EES-Gruppe bei einem Ohr (1/60 entspricht 2 %) eine Perforation vor. In der MES-Gruppe wies ein Patient eine Nekrose des tympanomeatalen Lappens mit einer Trommelfellperforation auf, 2 weitere Patienten Perforationen (3/60 entspricht 5 %), und ein Patient beklagte ein abstehendes Ohr nach retroaurikulärem Zugang.

## Diskussion

Die Verwendung des Endoskops anstelle des Mikroskops in der Mittelohrchirurgie hat sich an der Klinik der Autorin bewährt. Der Übergang vom Mikroskop zum Endoskop fällt leichter, wenn bereits Erfahrung mit der endoskopischen Nasennebenhöhlenchirurgie vorhanden ist. Assistenten in der Karlsruher Klinik erlernen während der Weiterbildung die endonasale Nasennebenhöhlenchirurgie. Nach der kombiniert mikroskopisch-endoskopischen Ausbildung im Felsenbeinlabor und Durchführung mindestens einer EES am Schafkopf werden zunächst Paukenröhrchen endoskopisch gelegt und dann Myringoplastiken und Tympanoplastiken Typ 1 durchgeführt [8].

Von den Patienten wird das minimal-invasive Verfahren und die Vermeidung eines äußerlichen Schnitts bevorzugt, wobei eine aktuell laufende klinische Studie hierzu noch nicht abgeschlossen ist. Es ist der Eindruck der Autorin und ihres Teams, dass die postoperativen Schmerzen nach einer EES geringer als nach einer MES sind, dies wurde in einer ersten Studie an einer anderen Klinik belegt [4].

Die vorliegende retrospektive Studie an 120 Patienten und prospektive Studien anderer Arbeitsgruppen zeigten, dass die Hörergebnisse der EES genauso gut wie die der MES sind [2, 3, 5, 6, 13, 14]. In der vorliegenden Studie gab es in der EES-Gruppe eine statistisch signifikante Verbesserung der Knochenleitung um −2 dB HL, die nicht erklärbar ist. Die postoperative Reduktion der Schallleitungsschwerhörigkeit (∆ ABG) und die Reduktion des Hörverlusts nach Röser 1973 (∆ HV) waren in beiden Gruppen statistisch signifikant nicht unterschiedlich (Tab. 3).

Zum Zeitpunkt der Detamponade sind Ohren nach EES tendenziell trockener und weiter in Abheilung begriffen als MES-Ohren. Dies ist vermutlich auf das geringere Weichteiltrauma beim endoskopischen Operieren zurückzuführen, wodurch weniger Wundsekret gebildet wird. In der MES-Gruppe wiesen 3 Patienten (5 %) bei Detamponade eine Perforation in der Paukenabdeckung auf im Gegensatz zu einem Patienten (2 %) in der EES-Gruppe. Möglicherweise ist dies darauf zurückzuführen, dass in der MES-Gruppe mehr ungeplante Tympanoplastikrevisionen (S2r = 20) als in der EES-Gruppe (S2r = 4) waren (Tab. 3).

Die kleinen Zahlen von sanierenden Ohreingriffen bei Adhäsivprozess oder Cholesteatom (19 in der EES-Gruppe, 12 in der MES-Gruppe) in der vorliegenden Studie erlauben keine valide Aussage über Residualcholesteatome und Rezidive, diesbezüglich wird auf die Literatur verwiesen [9, 12, 15]. In der vorliegenden Studie lag der Fokus auf den Schnitt-Naht-Zeiten, und hier ergab sich kein Unterschied zwischen den 2 Patientengruppen. In Bezug auf die Ergebnisse der Cholesteatomchirurgie fehlen zzt. noch valide Langzeitergebnisse aus prospektiven randomisierten Studien, um zu entscheiden, welches Verfahren besser ist, allerdings zeigen die ersten überwiegend retrospektiven Studien positive Ergebnisse [7, 9, 12, 15]. Die Hoffnung ist, dass durch die verbesserte endoskopische Visualisierung von Cholesteatommatrix in schwer einsehbaren Nischen des Mittelohrs die Ergebnisse im Vergleich zur mikroskopischen Cholesteatomchirurgie besser sein werden [15]. Hier steht der endgültige Beweis aber noch aus. Kleine epitympanale Cholesteatome lassen sich nach eigener Erfahrung sehr gut endoskopisch entfernen.

**Figure 3:**
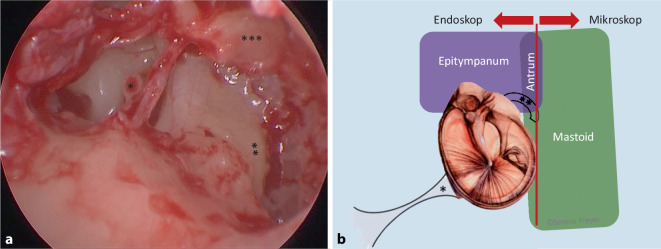

Ein großes Gegenargument gegen die Einführung der EES an einer Klinik ist die Sorge vor verlängerten Schnitt-Naht-Zeiten im Vergleich zur MES. In der vorliegenden Studie traf dies nicht zu (Abb. 2). Ein wichtiger Grund hierfür könnte die verbesserte Übersicht bei der EES sein, sodass seltener die vordere Gehörgangswand zurückgeschliffen werden musste; in den hier untersuchten 2 Patientenkollektiven war in 53/60 Fällen (88 %) bei der EES und nur in 24/60 Fällen (40 %) bei der MES keine Gehörgangserweiterung nötig. Darüber hinaus kann möglicherweise durch den transmeatalen Zugang zum Mittelohr bei der EES Zeit eingespart werden, welche bei der MES für Schnitt und Naht verlorengeht, wodurch das etwas langsamere Arbeiten im Mittelohr bei der EES kompensiert wird, sodass die Schnitt-Naht-Zeiten bei beiden Visualisierungstechniken gleich sind. Dieser zeiteinsparende Effekt würde bei langen Operationen in Relation zur Gesamtoperationszeit weniger ins Gewicht fallen. Es sollte daher bei Cholesteatomen und entzündeten Ohren präoperativ eine Computertomographie des Felsenbeins durchgeführt werden, um die Grenzen der pathologischen Veränderung bereits bei der Operationsplanung genau zu bestimmen. Bei ausgedehnten pathologischen Veränderungen, möglicherweise mit Bohrarbeit, sollte der mikroskopischen Vorgehensweise der Vorzug gegeben werden, da für die nötige Bohrarbeit das einhändige Arbeiten hinderlich ist und dann die Operationsdauer verlängert wird. Eine nach Erfahrung der Autorin sinnvolle Grenze stellen der laterale Bogengang und die Fossa incudis dar (Abb. 3). In Fällen mit Mastoidbefall kann nach Erfahrung der Autorin das Endoskop die MES ergänzen und beim Auffinden von kleinen Cholesteatomresten in schwer einsehbaren Nischen hilfreich sein. Während früher an der Klinik der Autorin bei Cholesteatomen mit Mastoidbefall ausschließlich mikroskopische A4-M2b- oder -M2c-Eingriffe (nach der SAMEO-ATO-Klassifizierung) durchgeführt wurden, wird dort nun gern eine kombinierte Vorgehensweise mit Beginn der Operation transmeatal endoskopisch A1-M2a zur Sanierung des Mittelohrs in Kombination mit retroaurikulär mikroskopisch A4-M1a oder -M1b zur Sanierung des Mastoids verwendet. Die hintere Gehörgangswand wird am Ende der Operation endoskopisch nach pathologischen Veränderungen abgesucht und kann häufiger als früher erhalten werden.

## Fazit für die Praxis

- Die endoskopischen Ohrchirurgie („endoscopic ear surgery“, EES) hat sich bei der Beseitigung von pathologischen Veränderungen des Mittelohrs bewährt.
- Die transmeatale Vorgehensweise ist minimal-invasiv, gewebeschonend und geht mit weniger postoperativen Schmerzen einher.
- Die Operationszeiten bei der EES unterscheiden sich statistisch nicht signifikant von der mikroskopischen Mittelohrchirurgie („microscopic ear surgery“, MES).
- Die Ergebnisse der EES in Bezug auf Trommelfellverschlussraten und Hörergebnisse gleichen der MES.
- Für die endoskopische Cholesteatomchirurgie steht die wissenschaftlich valide Bewertung noch aus.
- Erste Studien zeigen eine eher geringere Rezidivrate als bei der mikroskopischen Vorgehensweise.
